# Current Nomenclature of *Paracoccidioides lobogeorgii*’s Disease Name. Comment on Grotta et al. Fungal Density in Lobomycosis in French Guiana: A Proposal for a New Clinico-Histological and Therapeutic Classification. *J. Fungi* 2023, *9*, 1005

**DOI:** 10.3390/jof10010018

**Published:** 2023-12-28

**Authors:** Raquel Vilela, Leonel Mendoza

**Affiliations:** 1 Faculty of Farmacy, Federal University of Minas Gerais, Belo Horizonte 31270-901, Brazil; rrraquelvv@gmail.com; 2 Microbiology and Molecular Genetics, Michigan State University, East Lansing, MI 28824-1046, USA; 3 Biomedical Laboratory Diagnostics, Michigan State University, East Lansing, MI 28824-1046, USA

We have read the article by Grotta and collaborators [1] published in the *Journal of Fungi* about a new proposal for the clinical, pathological, and therapeutic classification of the disease caused by the uncultivable fungal pathogen *Paracoccidioides lobogeorgii* [2]. Since this etiologic agent was recently renamed from *Lacazia loboi* to *P. lobogeorgii* [2], the disease name should also follow proper nomenclatural rules. We will take the opportunity to highlight the taxonomic and nomenclatural changes currently in use, to correctly name the disease and the etiology of George Lôbo’s disease and a similar skin disease in dolphins now known as paracoccidioidomycosis ceti (PCMC) [3].

Ever since Jorge Lôbo in 1931 [4] first described a unique Brazilian human skin disease, its etiology and disease name have been at the center of endless nomenclatural and taxonomic controversies [5]. This in part was due to the fact that the yeast-like cells in the infected tissues resist culture and Jorge Lôbo’s failure to propose a name for its etiologic agent. The lack of a binomial for its etiology was the foundation leading to numerous genera, species, and disease names [2,5] (Table 1). One of the names in use for many years was the term “lobomycosis” [5,6]. This name was suggested by Borelli in 1958 as a provisional name [6]. In a one-page note, he proposed the genus *Lobomyces* without a Latin description and, thus, an invalid genus [6]. In the same note, he wrote that the disease described by Jorge Lôbo in humans “…*should be provisionally and concisely called lobomycosis, and its agent Lobomyces.*” [6]. Based on this premise, the term lobomycosis was considered by Lacaz [7] as an unacceptable disease name. Lacaz [7] wrote about the incorrect use of this terminology as follows: “*Regarding the disease caused by Paracoccidioides loboi* (currently *P. lobogeorgii*), *the correct disease name should be Jorge Lôbo’s disease and no lobomycosis. In Portuguese, lobo is the name of a wild animal*”. 

Lacaz’s proposal was ignored in part because of the taxonomic uncertainties surrounding the etiologic agent of Jorge Lôbo’s disease at that time, and thus, the term lobomycosis persisted. This trend continued even after 1999 when genus *Lacazia* (to honor Dr. Carlos da Silva Lacaz for his many contributions) was proposed [8]. Incidentally, in the title of the new proposed genus, Taborda et al. [8] overlooked Lacaz’s [5] arguments to avoid the use of Borelli’s disease name [6]. For the subsequent two decades, the genus *Lacazia* and the term lobomycosis remained in use [9,10] until Vilela et al. [2,3,11] relocated the etiologic agent of Jorge Lôbo’s disease to the genus *Paracoccidioides*. These authors reported that the DNA sequences extracted from infected dolphins with PCMC were different from the available DNA sequences from cases of Jorge Lôbo’s disease and, therefore, a different species. Before this study, both diseases were believed to be caused by the same etiologic agent; thus, the dolphin disease was also erroneously labelled with the name lobomycosis. 

The fundamental problem with the term “lobomycosis” resides in the fact that *P. lobogeorgii* is now part of the genus *Paracoccidioides* [2,3,11]. According to the disease name given to these pathogens (paracoccidioidomycosis), the need for a unique epithet to differentiate the fungal cultivable species causing systemic infection (*P. brasiliensis*, *P. americana*, and *P. lutzii*) from those uncultivable species causing only subcutaneous infections (*P. ceti* and *P. lobogeorgii*) is desirable. In addition, after the finding that the DNA sequences extracted from dolphins’ yeast-like cells were different from that in Jorge Lôbo’s human cases, the term lobomycosis in dolphins [12] is no longer a good choice for either disease. Therefore, to standardize the nomenclature of the diseases caused by the uncultivable *Paracoccidioides* species, the disease names Jorge Lôbo’s disease and paracoccidioidomycosis lobogeorgi (PCML), for the etiologic agent of *P. lobogeorgii*, and paracoccidioidomycosis ceti (PCMC), for the etiologic agent of *P. ceti*, in dolphins should be implemented [2,3]. Moreover, according to a recent literature review and population genetic analysis, Vilela et al. [2,11] stated that terms such as “*lobomycosis, lacaziosis, Lobos’s disease, and many others are no longer supported*” (Table 1). To standardize the terminology of fungal diseases, it is recommended that those working with *P. lobogeorgii* and *P. ceti* adhere to the current nomenclatural changes to avoid repetition of traditional mistakes.

Regarding the phylogenetic comparison between the DNA sequences of cases in other geographical areas with that from Brazil, mentioned by Grotta and collaborators [1], this information is available. Hornberger et al. [9] recently reported that the DNA sequences of a Guyana patient (geographic areas near the case reported by Grotta et al. [1]) with Jorge Lôbo’s disease (accession number MT112279) in a phylogenetic analysis clustered together with the DNA sequences of Brazilian Cases (accession numbers EU167500, EU167499, AF322182) and Mexican cases (accession numbers MN403304 and OR671231). We also know that DNA extracted from the infected tissues with yeast-like cells from an Italian man with Jorge Lôbo’s disease infected in Venezuela (accession number MH265101) [10] possessed the same DNA sequences as those reported in other Latin American human cases (unpublished data). These analyses suggest that *P. lobogeorgii* shared several DNA phylogenetic similarities among Latin American isolates tested so far. More specimens containing *P. lobogeorgii* yeast-like cells from different endemic areas need to be evaluated to validate these results. However, knowing the high level of speciation that exists in the cultivable *Paracoccidioides* species [13], phylogenetic differences among isolates of *P. lobogeorgii* and *P. ceti* could be expected.

Overall, it is important to remind authors dealing with these species to follow current nomenclatural and taxonomic rules, not only in the proper use of genera and species across a manuscript but also in adhering to the current nomenclatural changes, notably the misuse of the term “lobomycosis” that for years persisted in the literature. As Dr. Carlos da Silva Lacaz [7] once wrote, “……*the correct disease name should be Jorge Lôbo’s disease and no lobomycosis*”. 

## Figures and Tables

**Table 1:** Genera, species, and disease names used for the last 92 years for Jorge Lôbo’s disease [5].

Genera and Species Given to the Etiologic Agent of Jorge Lôbo’s Disease	Names Given to Jorge Lôbo’s Disease through the Years
*Glenosporella loboi*	Jorge Lôbo’s blastomycosis (1933)
*Glenosporopsis amazonica*	Blastomycosis Jorge Lôbo’s type (1935)
*Paracoccidioides loboi*	Jorge Lôbo’s disease * (1940)
*Blastomyces loboi*	Jorge Lôbo’s mycosis (1940)
*Loboa loboi*	Glenosporillosis (1946)
*Lobomyces loboi*	Keloid blastomycosis (1946)
*Lacazia loboi*	Keloidal blastomycosis (1946)
*Candida loboi*	Keloidal form of Lutz’s disease (1950)
*Paracoccidioides lobogeorgii* [2] *	Lôbo’s syndrome (1950)
	South American blastomycosis var. queloidiform (1950)
	Lôbo’s disease (1952)
	South American blastomycosis var. Jorge Lôbo (1955)
	Lobomycosis (1958)
	Jorge Lôbo’s keloidal blastomycosis (1959)
	Keloidiform blastomycosis (1963)
	Caibi’s leprosy (1966)
	Miriap-Piriap (1966)
	Lôbo’s blastomycosis (1969)
	Lôbo’s mycosis (1972)
	Lacaziosis (2006)
	Paracoccidiodomycosis lobogeorgi (PCML) [2] * (2023)

* Recognized genus and species and disease names.
